# Sex-specific vagal and spinal modulation of breathing with chest compression

**DOI:** 10.1371/journal.pone.0234193

**Published:** 2020-06-17

**Authors:** Alyssa Huff, Mitchell D. Reed, Kimberly E. Iceman, Dena R. Howland, Teresa Pitts

**Affiliations:** 1 Department of Physiology, University of Louisville, Louisville, KY, United States of America; 2 Department of Neurological Surgery, Kentucky Spinal Cord Injury Research Center, University of Louisville, Louisville, KY, United States of America; 3 Research Service, Robley Rex Veterans Affairs Medical Center, Louisville, KY, United States of America; National Yang-Ming University, TAIWAN

## Abstract

Lung volume is modulated by sensory afferent feedback via vagal and spinal pathways. The purpose of this study was to systematically alter afferent feedback with and without a mechanical challenge (chest compression). We hypothesized that manipulation of afferent feedback by nebulization of lidocaine, extra-thoracic vagotomy, or lidocaine administration to the pleural space would produce differential effects on the motor pattern of breathing during chest compression in sodium pentobarbital anesthetized rats (*N* = 43). Our results suggest that: 1) pulmonary stretch receptors are not the sole contributor to breathing feedback in adult male and female rats; 2) of our manipulations, chest compression had the largest effect on early expiratory diaphragm activity (“yield”); 3) reduction of spinally-mediated afferent feedback modulates breathing patterns most likely via inhibition; and 4) breathing parameters demonstrate large sex differences. Compared to males, female animals had lower respiratory rates (RR), which were further depressed by vagotomy, while chest compression increased RR in males, and decreased yield in females without changing RR. Collectively, our results suggest that balance between tonic vagal inhibition and spinal afferent feedback maintains breathing characteristics, and that it is important to specifically evaluate sex differences when studying control of breathing.

## Introduction

Lung volume is modulated by sensory afferent feedback transmitted through both vagal and spinal pathways. Vagal sensory feedback from pulmonary stretch receptors (PSRs) relays to the ventrolateral nucleus tractus solitarius (NTS) in the brainstem [1]. In respiratory muscles, such as intercostals and joint receptors of the ribcage, muscle spindles and Golgi tendon organs send proprioceptive information through spinal nerves into the spinal cord, which then ascends to respiratory centers in the medulla [2, 3].

PSRs respond to mechanical stimuli (tracheal/bronchial stretch) during lung inflation [4]. As the lungs expand, PSR activity increases, and drives pump cell activity in the NTS, ultimately resulting in inhibition of the ongoing inspiratory phase. During rapid inflation this is known as the Hering-Breuer Reflex [5, 6]. Vagotomy eliminates inputs from pulmonary afferents including mechanosensors (rapidly and slowly adapting receptors) and chemosensors (C-fiber and high-threshold Aδ-receptors) [7], it reduces respiratory rate (RR) by prolonging expiration, and it reduces variability in breath duration [6, 8–11].

Lung volume is also regulated by sensory feedback from proprioceptors in the thoracic cavity [3, 12]. Changes in chest wall volume and pressure provide information about muscle length-tension relationships [2, 12], and stretch receptors located in the thoracic cavity indirectly monitor lung inflation [3]. The primary function of the intercostal muscles is to mechanically maintain proper rib placement [13, 14] and to provide stability during movement. Their activity is modulated by feedback from muscle spindles, Golgi tendon organs, and other joint receptors [15–17]. Compared to caudal ribs, rostral ribs contain a higher density of mechanoreceptors and have more movement-related activation during breathing [15].

Vagotomy and chest compression have been studied in several species including dog, cat [18–20], rabbit [21, 22], and human [23, 24]. Vagotomy by itself generally decreases RR by increasing both inspiratory and expiratory durations [9–11, 25]. In humans, chest compression increases RR and reduces tidal volume [23]. Similarly, in one animal study, chest compression by itself increased RR by decreasing both inspiratory and expiratory durations, but this effect was abolished with vagotomy [21]. However, when animals are bilaterally vagotomized first, the addition of chest compression transiently inhibits phrenic inspiratory discharge and prolongs expiration for the initial breaths, but more sustained chest compression usually increases RR by decreasing both inspiratory and expiratory durations [18–20, 23, 26]. Collectively, these results suggest that vagotomy and chest compression have opposing effects on RR and inspiratory and expiratory durations, and that when the two conditions are combined, the individual effects may be counterbalanced by each other or additional influences. Thoracic proprioceptive feedback alters respiratory drive during loading (e.g. internal or external mechanical, elastic, or resistance loading). Work by Bolser, Shannon, and colleagues [27–32] demonstrates that activation of intercostal afferents (via Golgi tendon organs) strongly inhibits medullary inspiratory neurons, resulting in decreased diaphragm, intercostal, and laryngeal inspiratory muscle activity [29]. Inhaled airway anesthetics have been used to investigate the influence of airway receptors on ventilation, and human studies of inhaled lidocaine show inconsistent findings. The reports vary, with results suggesting that airway receptors play a major role in breathing at rest [33], during exercise but not at rest [34], or have no role in breathing during exercise [35], warranting further investigation. While bronchopulmonary vagal afferents and proprioceptive spinal afferents clearly have some influence on breathing, it is unclear how these afferents modulate the different phases of breathing. Manipulation of afferent feedback in each phase of breathing—inspiration and the subphases of expiration—allows for more detailed assessment of specific afferent effects.

The majority of physiology studies have traditionally only included males, and there are only a few studies on sex differences in respiration. These studies report that females have lower ventilation and higher RR than men under normal conditions [36], and lower partial pressure of carbon dioxide at rest [37]. Males and females also respond differently to ventilatory challenges [36]: female rats have a greater hypoxic ventilatory response than males at old age (>20 months) and a greater hypercapnic ventilatory response at middle age (12–13 months) [38]. Studies with humans suggest that women have a greater hypoxic ventilatory response than men, possibly due to high levels of progesterone (a known respiratory stimulant), but have a lower hypercapnic ventilatory response [39]. The relatively large number of investigations on sex differences in sleep apnea reveal that sleep apnea is more common in men than women, but its prevalence increases in women after menopause [40]. The use of hormone replacement therapy (estradiol and progesterone) in post-menopausal women decreases this prevalence [40]; low progesterone levels and increased occurrence of obstructive sleep apnea are correlated [41]. Females are thought to have better cardiorespiratory homeostasis and more neuroplasticity than males, resulting in greater tolerance to chronic intermittent hypoxia than males [41]. Little or no research has been dedicated to investigation of respiratory related sex differences during modulation of afferent feedback.

Respiration has the unique responsibility of maintaining gas exchange regardless of environmental demands and/or execution of other behaviors such as swallow. The full understanding of how the totality of afferent feedback alters respiratory regulation is unknown, particularly in the rat. Human studies on the role of airway receptors during exercise [35] and at rest produce conflicting results [33, 34]. Similar uncertainty about the ability of heart-lung transplant patients to maintain adequate ventilation and oxygenation levels during wakefulness and sleep [42, 43] invites further studies on specific effects of afferent feedback on breathing pattern. Additionally, the variability of the available data suggests there are unknown features that are important to its regulation (e.g. sex). The purpose of this study was to systematically alter afferent feedback before and during a mechanical challenge (chest compression) to further investigate afferent contribution to breathing patterns. We tested the hypothesis that selective inhibition of PSRs, vagotomy, or lidocaine administration to the pleural space would produce different effects on breathing during chest compression. Additionally, we predicted that females would have a more pronounced response, due to less advantageous chest wall geometry and a higher contribution of the chest wall to breathing at rest [44, 45].

## Methods

Experiments were performed on 48 anesthetized spontaneously breathing Sprague Dawley (SD) retired breeder rats [24 male (0.49 ± 0.04kg) and 19 female (0.39 ± 0.08kg) Envigo, Indianapolis, IN], of which only 43 completed the protocol. Ages ranged from 8–9 months, which were shared with a complementary paper to this study [46]. We recognize that retired breeders are older than the general adult rat, however studies investigating breathing and aging use SD rats with an average age of 13 months [47]. The protocols were approved by University of Louisville Institutional Animal Care and Use Committee (IACUC). The animals were initially anesthetized with gaseous isoflurane (1.5–2% with 100% O_2_) using a head cone while a femoral intravenous (i.v.) cannula was placed for administration of sodium pentobarbital (25 mg/kg, i.v.). Isoflurane was discontinued and supplementary doses of sodium pentobarbital were administered as needed throughout the experiment. Anesthetic level was evaluated by withdrawal reflex of the forelimb and hindlimb and licking in response to oral water administration. A dose of atropine sulfate (0.01mg/kg, i.v.) was given at the beginning of the experiment to reduce secretions from repeated tracheal stimulation. Following administration of atropine sulfate, a tracheostomy was performed and followed by incision of the esophagus for placement of a 20 gauge catheter to measure esophageal pressure. Body temperature was maintained using a heating pad. After completion of the experimental protocol, euthanasia was induced by an overdose of sodium pentobarbital followed by either administration of Beuthanasia D (Merck Animal Health) or potassium chloride.

Electromyograms (EMG) of multiple respiratory-related muscles were recorded using bipolar insulated fine wire electrodes according to the technique of Basmajian and Stecko [48]. The costal diaphragm (sternal) and the thyroarytenoid muscle (primary laryngeal adductor) were used to evaluate breathing. The thyroarytenoid electrodes were inserted through the cricothyroid window into the anterior portion of the vocal folds. Electrode placements were visually inspected and verified post-mortem. For electrode placement of the costal diaphragm, palpation and elevation of the xyphoid process was followed by insertion of a needle directed caudally. The needle was hooked underneath the xyphoid process near the costal diaphragm muscle attachment. Electrodes were placed bilaterally into the pectoralis muscle to record electrocardiogram (ECG) activity used to remove heart artifact from EMG traces.

### Defining respiratory phase

**Fig 1:**
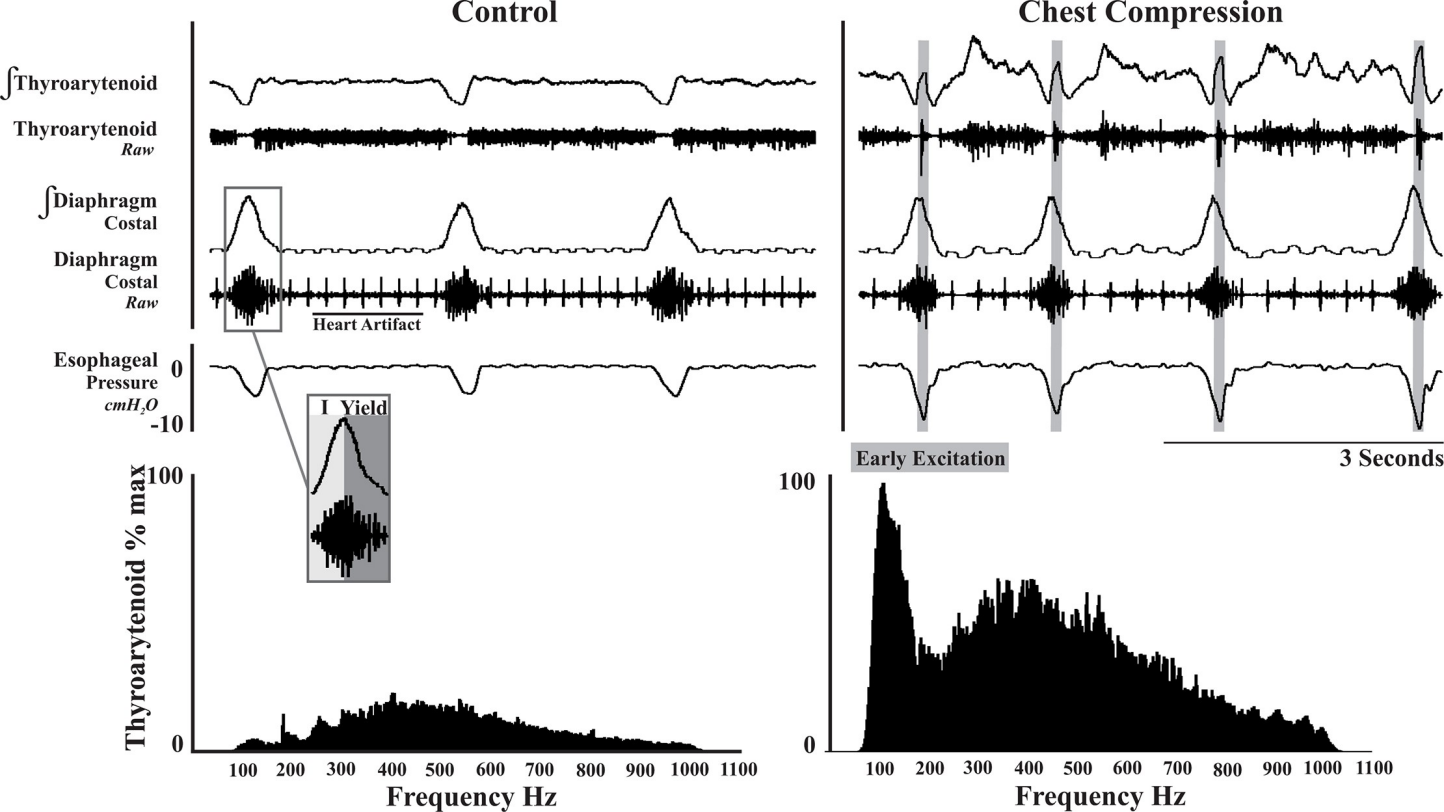
E1 thyroarytenoid activity during control conditions (eupnea) and chest compression. (Top) Integrated and raw EMG traces of corresponding muscle activity and esophageal pressure show the activity change in the muscle pattern and esophageal pressure change when chest compression is applied. The grey vertical rectangles represent the early activation of the thyroarytenoid (laryngeal adductor) muscle during expiratory yield. (Bottom) The power spectrum analysis illustrates an increase in EMG amplitude with chest compression, and an early burst of laryngeal adductor activity during the yield phase of breathing.

**Fig 2:**
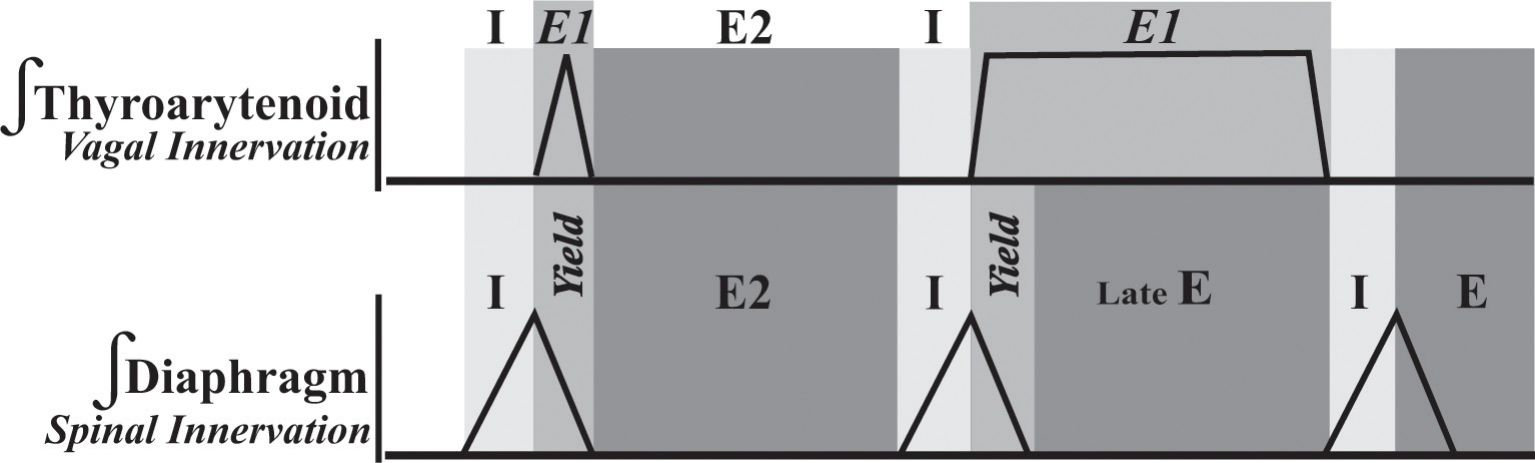
Diagram of proposed subcomponents of expiration with divergent vagal (thyroarytenoid, E1) and spinal mechanisms (diaphragm, yield). Diaphragm quiet breathing activity shows two phases: inspiration (I) (onset of diaphragm activity to peak diaphragm activity) and expiration (E) (peak of diaphragm activity to the onset of subsequent diaphragm activity). The subphases of expiration are defined as: early expiration (E1) (vagal efferent activation of the thyroarytenoid muscle: laryngeal adductor), yield (the spinal inspiratory muscle activity that remainins during early expiration), late E (inactivity of diaphragm during thyroarytenoid activity), and E2—true passive expiration—(beginning at the offset of thyroarytenoid activity and lasting until the onset of the next diaphragm activation). E1 represents active contraction of the thyroarytenoid muscle during expiration and E2 is the passive movement of lung recoil. In the present study, we observed only the thyroarytenoid E1 phase, and no E2 phase, as the thyroarytenoid muscle contracted at the peak activation of the diaphragm and relaxed at the onset of the next diaphragm activation.

In the present study, inspiration (I) was defined as the period from the onset of diaphragm activity to the peak of the diaphragm burst, and expiration as the period from the peak of diaphragm activity to the onset of subsequent diaphragm activity (Figs 1 and 2). We defined vagal efferent activity that begins in early expiration as E1 (i.e. thyroarytenoid: laryngeal adductor), and the spinal inspiratory muscle activity that remains during early expiration as “yield”. Late expiration (late E) was defined as the period from the offset of diaphragm activity to the onset of subsequent diaphragm activity (Figs 1 and 2).

### Experimental protocol

Three experimental protocols were performed on three cohorts of male and female SD rats. A) An extra-thoracic vagotomy was performed in 12 rats [6 male (0.48 ± 0.03kg) and 6 female (0.35 ± 0.06kg)]. B) Lidocaine (10%) was nebulized into the trachea in 18 rats [8 male (2 sham) (0.40 ± 0.03kg), 10 female (2 sham)]. Only 5 of these females (2 of which are sham) completed the protocol (0.39 ± 0.09kg) and are included in the data presented. C) Lidocaine (10%) was injected into the pleural space in 18 rats [10 male (2 sham) (0.46 ± 0.05kg), 8 female (2 sham) (0.41 ± 0.08kg)]. In sham experiments for protocol B), saline (diluent) was nebulized into the trachea; for protocol C), saline was injected into the pleural space.

### Removal/Reduction of vagal feedback

A) To remove all vagal afferent feedback, bilateral vagotomy at the level of the extra-thoracic trachea was performed on rats in the supine position. The vagus nerves were dissected away from the sympathetic nerves and common carotid arteries. Silk suture (5–0) was looped around each vagus nerve. Hemostat forceps were clamped onto the suture ends for quick access after control trials had been completed. At the appropriate time, the suture attached to the hemostats was lifted so that the vagus nerves could be cut using spring scissors at the level of the 5^th^– 6^th^ tracheal ring. After bilateral vagotomy, an inflation test was performed to ensure removal of PSR (lung volume) feedback: 4 cc of air was drawn into a 5 cc syringe and quickly infused into the endotracheal tube. The order of the cuts were randomized (left vs right) across animals.

B) To selectively reduce vagal feedback from PSRs, 10% lidocaine was nebulized into the trachea [49–51] with the animal in the supine position. Using a compressor nebulizer (Strong Health; particle size 0.5–5μm; average nebulization rate 0.2 mL/min), 10% Lidocaine (Cat No. L5647, Sigma-Aldrich) mixed with 2% Evans Blue Dye (EBD, Cat No. E2129, Sigma-Aldrich) was nebulized for 15 minutes. Ten minutes after the completion of the nebulization, we performed an inflation test by injecting 4 cc of air into the trachea. If the Hering-Breuer reflex was maintained (i.e. termination of inspiration followed by prolonged expiration), suggesting that PSRs were not anesthetized, the animal then received an additional 5 minutes of nebulized lidocaine and was retested. This procedure was performed as necessary until the reflex was abolished. The addition of the dye allowed for post-mortem verification that the lidocaine penetrated the lung tissue and the intra- and extra-thoracic trachea. To minimize contamination of the lidocaine and dye into the air and to the researcher, a portable fume evacuation machine hovered over the mouthpiece of the nebulizer. To minimize contamination around the trachea, Vaseline-coated gauze was placed below and above the trachea, which covered any exposed area of the animal and blocked any potential absorption of lidocaine into the upper airway that was not specifically targeted by nebulized lidocaine.

### Reduction of spinal feedback

C) To reduce spinal feedback, bilateral injections of 10% lidocaine mixed with 2% EBD were administered into the pleural space using methods from Mantilla et al. [52], which labeled motoneurons in both the cervical and thoracic segments indicating phrenic and intercostal innervation [52]. Animals were stabilized on their sides while the rib cage was palpated to identify the fifth intercostal space. The injection site was located one inch rostral to the xyphoid process and lateral to the sternum where the axial portion of the rib lies and then marked with a permanent marker. This was repeated on the other side. At this location the skin was removed using skin scissors, and 20μl of lidocaine/EBD mixture was injected bilaterally using a 100-μl Hamilton syringe with a 35 gauge beveled needle inserted 6 mm so that it reached into the pleural cavity. After both injections were complete, the animal was returned to supine position, and after a 5 minute waiting period an inflation test was performed to confirm that a reflex response was present, indicating that the lidocaine had not affected the PSRs or altered any other vagal afferent feedback.

#### Stimuli

Chest compression stimuli were performed during control conditions (before lidocaine or vagotomy interventions) and after interventions. Chest compression of the thoracic cavity was performed by placing a 2-inch thick circumferential Velcro band around the chest to restrict movement to the end of expiration tidal volume. In order to monitor movement of the chest wall, a custom in-house produced chest strap was made using a piezoelectric sensor from a fire alarm, an aluminum plate, and an elastic band (1/2 inch). The sensor was mounted on the aluminum plate, which was loosely strapped around the animal’s chest rostral to the Velcro restriction band. This piezoelectric chest strap allowed us to observe the change in movement resulting from the restrictive band. Video was captured for visual verification of the reduction in chest movement.

### Analysis

All EMG signals were amplified and filtered (100–1000 Hz) using Grass P511 (Natus Neurology) amplifiers. Esophageal pressure was measured by a TA-100 single channel transducer amplifier (CWE, Inc). Signals were rectified and integrated (20ms) using Spike2 (Cambridge Electronic Design; Cambridge, England). EMG amplitude measures were calculated as a percent of maximum during the control period. Breathing phase durations were measured using diaphragm EMG activity across 30 seconds of eupnea. Eupneic periods during the control period and the control chest compression conditions were averaged separately for male (n = 24) and female (n = 19) groups. Respiratory rate (RR) was calculated as the number of respiratory cycles during a 30 second period, multiplied by 2. To normalize the signal across animals, EMG amplitude data is presented as a percent of the maximum from the control period.

Results are expressed as means ± standard deviation (SD). Paired and independent t-tests and 2-way ANOVA were used as appropriate to statistically identify differences using SPSS statistical software (IBM Corporation). Analyses were made within groups (male and female) and between groups (male vs female). A difference was considered significant if the *p-*value was less than 0.05.

## Results

Significant changes are described below; all data are presented in Tables 1 and 2.

**Table 1:** Means, standard deviation (SD), *p-*values, and direction of change for breathing parameters during control and chest compression conditions for both male and female groups. Diaphragm amplitude is normalized to maximum of control and shown as a percentage. Respiratory rate (RR) was calculated as number of cycles within a 30 second period multiplied by 2. Reported *p-*values are from Student’s paired t-test. Significance is bolded at *p-*values ≤ 0.05 and *p-*values indicating trends towards significant of 0.05 < x ≤ 0.07 are *italicized*.

	Control				Chest Compression					
	mean	(	SD	)	mean	(	SD	)	*p*-value	Change
**Male (*n* = 24)**		** **	** **	** **	** **	** **	** **	** **	** **	
Diaphragm Amplitude (% max)	78	(	7	)	82	(	36	)	0.63	**-**
Inspiration Duration (ms)	122	(	66	)	117	(	42	)	0.46	**-**
Yield Duration (ms)	99	(	48	)	89	(	41	)	*0*.*06*	*↓*
Late Expiration Duration (ms)	604	(	172	)	518	(	152	)	**0.006**	**↓**
Thyroarytenoid Duration (ms)	604	(	194	)	556	(	165	)	0.18	**-**
Total Respiratory Cycle (ms)	824	(	163	)	720	(	172	)	**0.002**	**↓**
RR	74	(	13	)	82	(	17	)	**0.007**	**↑**
**Female (*n* = 19)**		** **	** **	** **	** **	** **	** **	** **	** **	
Diaphragm Amplitude (% max)	69	(	17	)	91	(	43	)	**0.03**	**↑**
Inspiration Duration (ms)	132	(	60	)	125	(	42	)	0.54	**-**
Yield Duration (ms)	119	(	65	)	82	(	29	)	**0.01**	**↓**
Late Expiration Duration (ms)	694	(	394	)	670	(	361	)	0.65	**-**
Thyroarytenoid Duration (ms)	782	(	457	)	756	(	446	)	0.73	**-**
Total Respiratory Cycle (ms)	936	(	384	)	878	(	370	)	0.28	**-**
RR	70	(	19	)	77	(	26	)	0.19	**-**

**Table 2:** Means, standard deviation (SD), *p-*values and direction of change for breathing parameters during conditions of control (no feedback modulation or chest compression), feedback modulation alone (e.g. vagotomy), chest compression alone (without feedback modulation), and chest compression (CC) during feedback modulation conditions for both male and female groups. The left half of the table shows data comparing control conditions (no feedback modulation) to conditions adding vagotomy (A), nebulized lidocaine (B), or pleural injection of lidocaine (C), while the right half compares chest compression with the addition of each intervention. Diaphragm amplitude is normalized to maximum of control and shown as a percentage. Respiratory rate (RR) was calculated as number of cycles within a 30 second period multiplied by 2. Reported *p-*values are from Student’s paired t-test. Significance is **bolded** at *p-*values ≤ 0.05 and *p-*values of 0.05 < x ≤ 0.07 indicating trends are *italicized*.

A		Control				Vagotomy						Chest Compression				CC+Vagotomy					
		mean	(	SD	)	mean	(	SD	)	*p*-value	Change	mean	(	SD	)	mean	(	SD	)	*p*-value	Change
	**Male (*n* = 6)**		** **	** **	** **	** **	** **	** **	** **	** **		** **	** **	** **	** **	** **	** **	** **	** **	** **	
	Diaphragm Amplitude (% max)	80	(	4	)	117	(	53	)	0.13	**-**	69	(	22	)	97	(	54	)	0.16	**-**
	Inspiration Duration (ms)	134	(	37	)	184	(	38	)	*0*.*06*	↑	117	(	14	)	211	(	106	)	0.08	**-**
	Yield Duration (ms)	111	(	28	)	132	(	37	)	0.14	**-**	93	(	16	)	147	(	31	)	**0.002**	**↑**
	Late Expiration Duration (ms)	622	(	152	)	538	(	275	)	0.49	**-**	454	(	167	)	368	(	180	)	0.10	**-**
	Thyroarytenoid Duration (ms)	-	(	-	)	-	(	-	)	-	**-**	-	(	-	)	-	(	-	)	-	**-**
	Total Respiratory Cycle (ms)	837	(	141	)	850	(	300	)	0.91	**-**	652	(	196	)	695	(	204	)	0.30	**-**
	RR	73	(	12	)	75	(	19	)	0.74	**-**	86	(	15	)	81	(	12	)	0.37	**-**
	**Female (*n* = 6)**		** **	** **	** **	** **	** **	** **	** **	** **		** **	** **	** **	** **	** **	** **	** **	** **	** **	
	Diaphragm Amplitude (% max)	72	(	15	)	129	(	78	)	0.11	**-**	78	(	37	)	171	(	119	)	0.11	**-**
	Inspiration Duration (ms)	124	(	72	)	274	(	100	)	*0*.*07*	↑	107	(	15	)	221	(	70	)	**0.02**	**↑**
	Yield Duration (ms)	99	(	29	)	162	(	92	)	0.13	**-**	78	(	14	)	154	(	50	)	**0.02**	**↑**
	Late Expiration Duration (ms)	849	(	408	)	1245	(	444	)	**0.02**	**↑**	864	(	319	)	837	(	220	)	0.89	**-**
	Thyroarytenoid Duration (ms)	-	(	-	)	-	(	-	)	-	**-**	-	(	-	)	-	(	-	)	-	**-**
	Total Respiratory Cycle (ms)	1050	(	357	)	1647	(	489	)	**0.01**	**↑**	1047	(	325	)	1185	(	283	)	0.51	**-**
	RR	62	(	22	)	40	(	15	)	**0.04**	**↓**	61	(	17	)	52	(	11	)	0.33	**-**
**B**	** **	**Control**				**Nebulize**			** **	** **		**C****hest** **C****ompression**			** **	**CC+Nebulize**			** **	** **	** **
	** **	**mean**	**(**	**SD**	**)**	**mean**	**(**	**SD**	**)**	***p*-value**	**Change**	**mean**	**(**	**SD**	**)**	**mean**	**(**	**SD**	**)**	***p*-value**	**Change**
	**Male (*n* = 6)**		** **	** **	** **	** **	** **	** **	** **	** **		** **	** **	** **	** **	** **	** **	** **	** **	** **	
	Diaphragm Amplitude (% max)	76	(	12	)	108	(	40	)	0.09	**-**	78	(	44	)	117	(	58	)	**0.01**	**↑**
	Inspiration Duration (ms)	88	(	13	)	226	(	291	)	0.32	**-**	85	(	22	)	111	(	31	)	0.21	**-**
	Yield Duration(ms)	79	(	17	)	81	(	27	)	0.91	**-**	73	(	17	)	85	(	36	)	0.55	**-**
	Late Expiration Duration (ms)	663	(	129	)	530	(	241	)	0.20	**-**	509	(	97	)	588	(	222	)	0.50	**-**
	Thyroarytenoid Duration (ms)	619	(	156	)	539	(	260	)	0.70	**-**	476	(	266	)	586	(	195	)	0.60	**-**
	Total Respiratory Cycle (ms)	824	(	131	)	831	(	375	)	0.97	**-**	661	(	104	)	782	(	241	)	0.38	**-**
	RR	74	(	10	)	89	(	52	)	0.54	**-**	85	(	25	)	74	(	18	)	0.46	**-**
	**Female (*n* = 3)**		** **	** **	** **	** **	** **	** **	** **	** **		** **	** **	** **	** **	** **	** **	** **	** **	** **	
	Diaphragm Amplitude (% max)	74	(	14	)	91	(	40	)	0.40	**-**	117	(	15	)	137	(	70	)	0.71	**-**
	Inspiration Duration (ms)	130	(	48	)	130	(	7	)	0.99	**-**	148	(	44	)	141	(	22	)	0.75	**-**
	Yield Duration (ms)	79	(	34	)	79	(	21	)	0.93	**-**	62	(	11	)	87	(	38	)	0.28	**-**
	Late Expiration Duration (ms)	639	(	169	)	733	(	165	)	0.61	**-**	442	(	234	)	499	(	187	)	0.78	**-**
	Thyroarytenoid Duration (ms)	-	(	-	)	-	(	-	)	-	**-**	-	(	-	)	-	(	-	)	-	**-**
	Total Respiratory Cycle (ms)	839	(	123	)	942	(	150	)	0.51	**-**	652	(	209	)	727	(	202	)	0.72	**-**
	RR	70	(	12	)	63	(	10	)	0.60	**-**	99	(	41	)	89	(	29	)	0.80	**-**
**C**	** **	**Control**				**Pleural Injection**			** **	** **		**C****hest** **C****ompression**				**CC+Pleural Injection**				** **	
	** **	**mean**	**(**	**SD**	**)**	**mean**	**(**	**SD**	**)**	***p*-value**	**Change**	**mean**	**(**	**SD**	**)**	**mean**	**(**	**SD**	**)**	***p*-value**	**Change**
	**Male (*n* = 8)**		** **	** **	** **	** **	** **	** **	** **	** **		** **	** **	** **	** **	** **	** **	** **	** **	** **	
	Diaphragm Amplitude (% max)	78	(	8	)	73	(	36	)	0.73	**-**	99	(	40	)	94	(	44	)	0.47	**-**
	Inspiration Duration (ms)	147	(	104	)	169	(	78	)	0.58	**-**	146	(	57	)	150	(	60	)	0.84	**-**
	Yield Duration (ms)	123	(	71	)	126	(	26	)	0.88	**-**	105	(	64	)	125	(	87	)	0.08	**-**
	Late Expiration Duration (ms)	614	(	251	)	468	(	171	)	**0.03**	**↓**	597	(	188	)	545	(	205	)	0.31	**-**
	Thyroarytenoid Duration (ms)	642	(	221	)	508	(	36	)	0.30	**-**	644	(	114	)	598	(	88	)	0.30	**-**
	Total Respiratory Cycle (ms)	880	(	220	)	758	(	129	)	0.09	**-**	848	(	183	)	820	(	165	)	0.52	**-**
	RR	71	(	17	)	81	(	13	)	0.10	**-**	73	(	13	)	76	(	15	)	0.39	**-**
	**Female (*n* = 6)**		** **	** **	** **	** **	** **	** **	** **	** **		** **	** **	** **	** **	** **	** **	** **	** **	** **	
	Diaphragm Amplitude (% max)	64	(	25	)	110	(	51	)	0.10	**-**	97	(	62	)	182	(	111	)	**0.05**	**↑**
	Inspiration Duration (ms)	142	(	49	)	237	(	134	)	*0*.*06*	↑	124	(	30	)	186	(	53	)	**0.02**	**↑**
	Yield Duration (ms)	160	(	60	)	133	(	72	)	0.48	**-**	98	(	43	)	126	(	93	)	0.31	**-**
	Late Expiration Duration (ms)	713	(	572	)	533	(	427	)	0.14	**-**	638	(	457	)	353	(	277	)	**0.03**	**↓**
	Thyroarytenoid Duration (ms)	874	(	759	)	845	(	503	)	0.90	**-**	765	(	698	)	605	(	390	)	0.40	**-**
	Total Respiratory Cycle (ms)	1004	(	579	)	902	(	429	)	0.29	**-**	857	(	511	)	651	(	294	)	0.10	**-**
	RR	72	(	25	)	75	(	24	)	0.52	**-**	81	(	25	)	103	(	32	)	**0.03**	**↑**

### Chest compression changes early expiratory activity (Table 1)

When compared to control conditions, chest compression decreased yield duration in females (Table 1), but also changed the pattern of thyroarytenoid (laryngeal adductor) muscle activation (Fig 1). Comparing chest compression to control eupnea, female rats demonstrated a 22% increase in diaphragm EMG activity (*p* < 0.03), and a significant decrease in yield (early expiratory EMG activity) duration. In males: late E duration significantly decreased (604 ± 172ms to 518 ± 152ms, t_23_ = 3.0, *p <* 0.01), and total respiratory cycle (TRC) duration significantly decreased (824 ± 163ms to 720 ±172ms, t_23_ = 3.6, *p <* 0.01); this resulted in a significant increase in RR (74 ± 13 to 82 ±17, t_23_ = -3.0, *p <* 0.01). The power spectra in Fig 1 illustrate an increase in EMG amplitude with chest compression, and an early burst of laryngeal adductor activity during the yield phase of breathing.

### Extra-thoracic vagotomy

After vagotomy (Table 2A), female animals had a significant increase in late E duration (849 ± 408ms to 1245 ± 444ms, t_5_ = -3.5, *p <* 0.02), which increased TRC (1050 ± 357ms to 1647 ± 489ms, t_5_ = -3.8, *p <* 0.02) and decreased RR (62 ± 22 to 40 ± 15, t_5_ = 2.7, *p <* 0.05); this was not true for male animals. There also was a trend towards increase in I duration for males (134 ± 37ms to 184 ± 38ms, t_5_ = -2.5, *p =* 0.06) and females (124 ± 72ms to 274 ± 100ms, t_5_ = -2.3, *p =* 0.07); data are not shown, as all other measures were not significantly different.

With the addition of chest compression (Table 2A), vagotomized female animals had a significant increase in I duration (107 ± 15ms to 221 ± 70ms, t_5_ = -3.5, *p <* 0.02). Yield (early expiratory activity) duration increased in both males (93 ± 16ms to 147 ± 31ms, t_5_ = -5.9, *p <* 0.01) and females (78 ± 14ms to 154 ± 50ms, t_5_ = -3.4, *p =* 0.02).

### Nebulized lidocaine

After lidocaine nebulization (Table 2B), there was no change in diaphragm amplitude, I, yield, late E, TRC, or RR in either males or females. Female sham animals had a significant decrease in late E duration (527 ± 110ms to 439 ± 105ms, t_1_ = 25.7, *p =* 0.03).

In male animals with chest compression (Table 2B), the percent of maximum diaphragm EMG amplitude increased by 39% (*p =* 0.01) following nebulization of lidocaine.

### Altered afferent feedback by pleural administration of lidocaine

After injection of lidocaine into the pleural space (Table 2C), females had a non-significant increase in I duration (142 ± 49ms to 237 ± 134ms, t_5_ = -2.4, *p* = 0.06, Table 2C), while males had a significant decrease in late E duration (614 ± 251ms to 468 ± 171ms, t_5_ = 2.8, *p <* 0.03).

In female animals with chest compression (Table 2C), pleural lidocaine administration produced an 85% increase in diaphragm EMG amplitude (*p <* 0.05), an increase in I duration (124 ± 30ms to 186 ± 53ms, t_5_ = -3.3, *p <* 0.05), and a decrease in late E duration (638 ± 457ms to 353 ± 277ms, t_5_ = 3.2, *p <* 0.05); an increase in RR was also seen (81 ± 25 to 103 ± 32, t_5_ = -3.0, *p <* 0.05).

### Sex differences

In control conditions, female animals had significantly longer late E durations (694 ± 394ms to 604 ± 172ms, F_1_ = 4.0, *p =* 0.05), thyroarytenoid EMG durations (782 ± 457ms to 604 ± 194ms, F_1_ = 5.7, *p =* 0.02), TRC durations (936 ± 384ms to 824 ± 163ms, F_1_ = 4.9, *p =* 0.03), and a slower RR (70 ± 19 to 74 ± 13, F_1_ = 2.7, *p =* 0.02) compared to males. Vagotomy enhanced these differences. Late E durations (1245 ± 444ms to 538 ± 275ms, F_1_ = -3.3, *p =* 0.01) and RRs (40 ± 15 to 75 ± 19, F_1_ = 3.5, *p <* 0.01) were greater in females than males in vagotomized animals without chest compression (late E duration: 1245 ± 444ms to 538 ± 275ms, F_1_ = -3.3, *p =* 0.01; RR: 40 ± 15 to 75 ± 19, F_1_ = 3.5, *p <* 0.01), and with the addition of chest compression (late E durations: 837 ± 220ms to 368 ± 180ms, F_1_ = -4.0, *p <* 0.01; RR: 52 ± 11 to 81 ± 12, F_1_ = 4.6, *p <* 0.01).

### Poincaré plots of breathing phase durations

**Fig 3:**
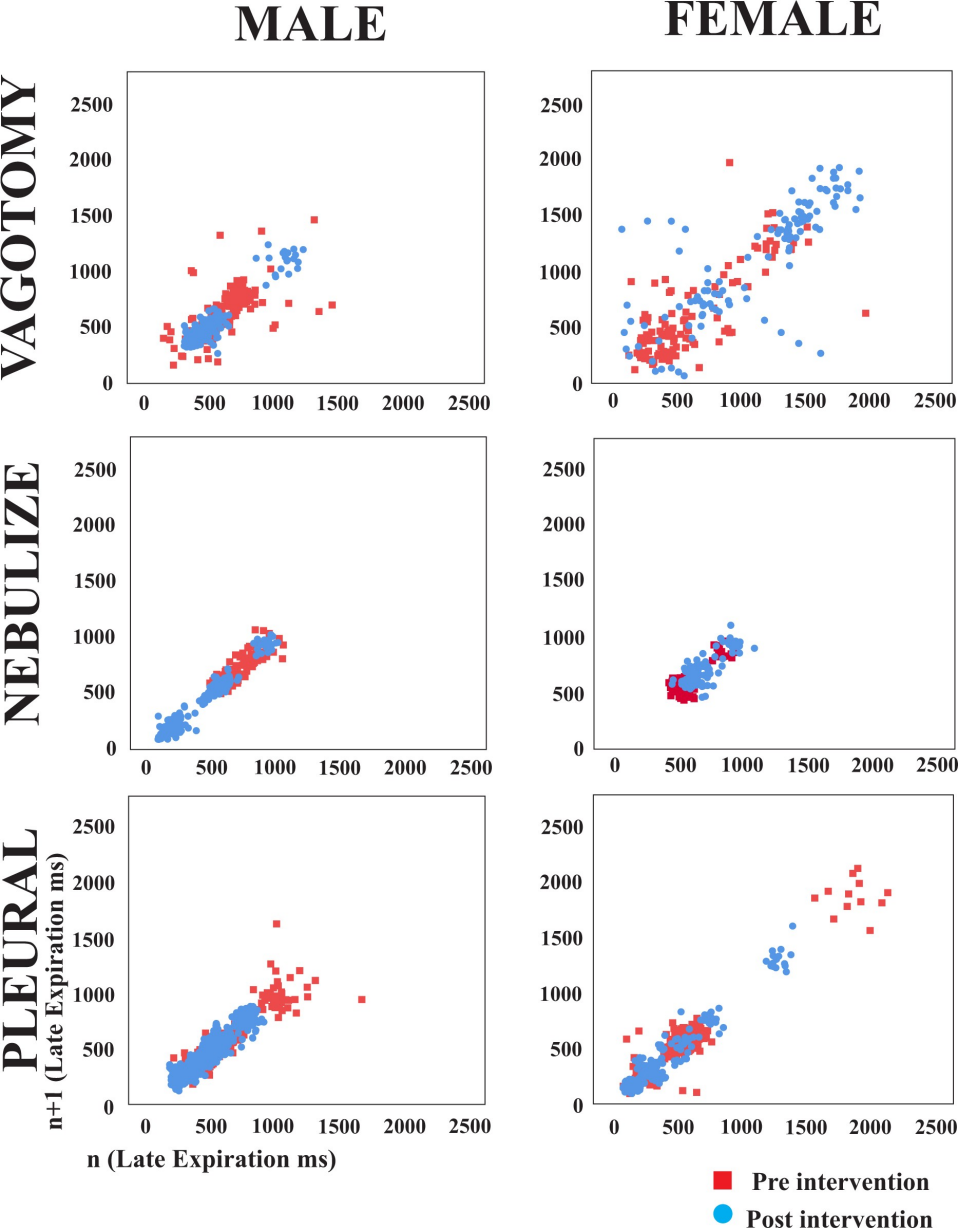
Poincaré plots illustrate difference in late expiration duration variability across afferent feedback interventions in both male and female animals. The red squares represent control conditions and the blue circles represent post-intervention conditions. In male animals, the points are more tightly clustered after vagotomy and pleural injection interventions.

Fig 3 displays Poincaré plots of the late E duration variability over 30 second periods for the different afferent feedback interventions. The points are tightly clustered for post-vagotomy males, indicating low variability, while the more dispersed points for the vagotomized females illustrate the opposite effect. When lidocaine was nebulized, there was no change in the tightness of the clustering, but the entire cluster shifted to the left (indicating a decrease in late E duration) in males; in females the cluster shifted to the right. When spinal feedback was reduced with pleural lidocaine injections, there was a slight decrease in variability in males and females, as indicated by greater clustering of points.

### Variability

**Fig 4:**
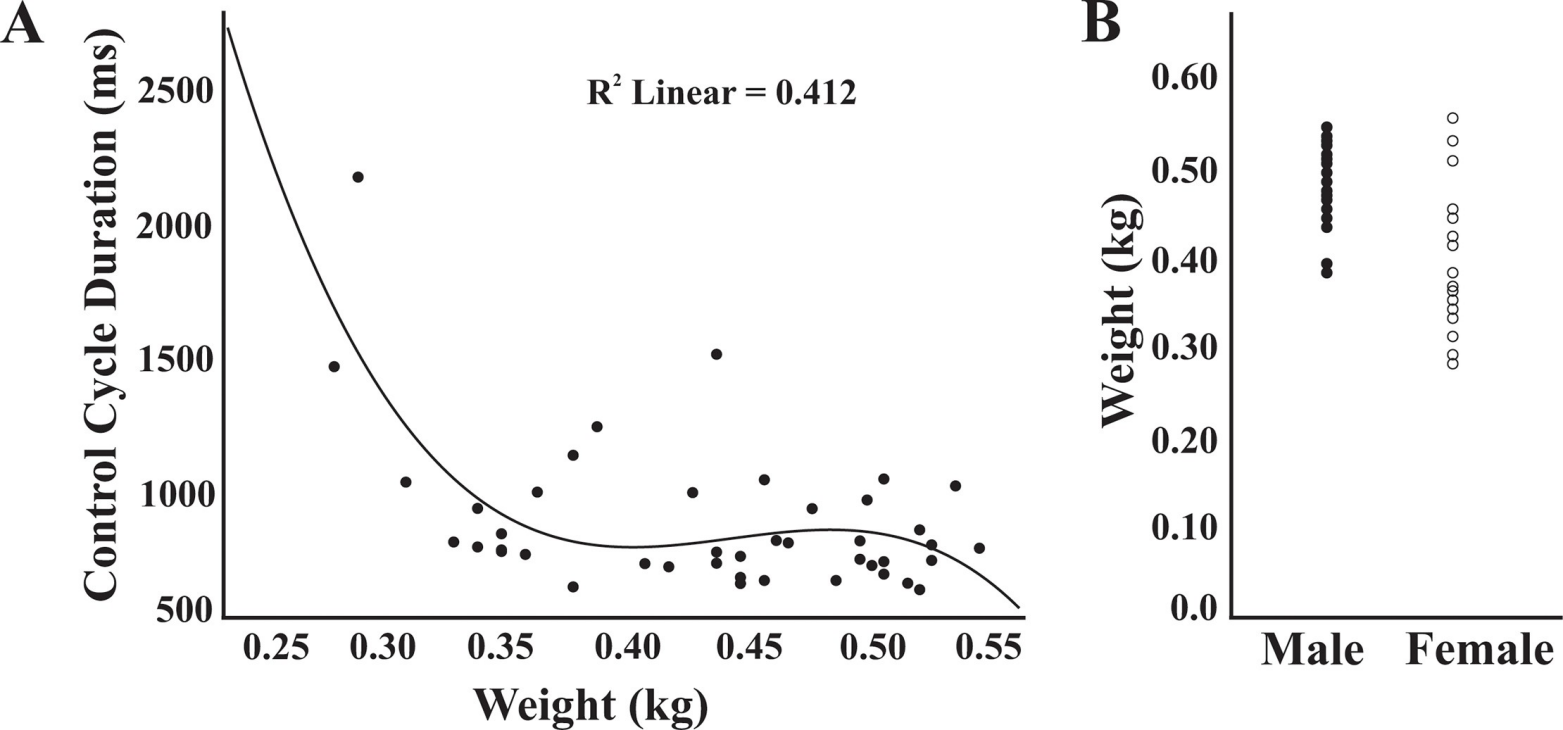
Scatter plots showing A) distribution of control breathing cycle duration versus weight and B) weight distribution of male and female animals. These scatter plots show the large variability of female rat weights compared to male weights.

For all animals, control TRC was plotted against animal weight, with a resulting *R*^*2*^ value of 0.41 (Fig 4A). Fig 4B displays weight of each animal plotted against sex, and shows that the females had the largest variance and spanned the smallest to the largest weight.

## Discussion

The goal of this study was to evaluate breathing during a mechanical challenge, while systematically altering spinal and vagal afferent feedback. Mechanical challenge was achieved by restricting chest wall movements (via banding), along with performing vagotomy, selectively anesthetizing PSRs, or reducing spinal feedback from pleural afferents. The present results demonstrate that: a) the classic respiratory response to vagotomy is not solely due to eliminating PSR feedback, b) there is a balance in vagal and spinal feedback that alters remnant diaphragm activity in early expiration (i.e. yield; Fig 1), and c) injection of lidocaine into the pleural space modulates breathing, most likely by inhibiting feedback from pleural spinal afferents.

### Chest compression

External pressure to the thoracic cavity was first applied by Adrian [53], who reported an increase in RR and vagal feedback. The RR effects have been replicated in humans and dogs [23], cats and dogs [19], and rabbits [21]. As we hypothesized, chest compression increased RR, but the increase was statistically significant only in male animals. Of note, increased RR is often a result of hypercapnia (respiratory acidosis due to insufficient alveolar ventilation). Though we did not directly measure blood gases during this study, other work indicates that the increases in RR due to chest compression are not a direct result of increased chemical drive in either intact or vagotomized animals [18] (in contrast to an elastic load [19]). Shannon attributed the alteration in RR to stimulation of chest wall mechanoreceptors [19].

### Yield: A novel description of diaphragm activity during early expiration

The classic definition of yield is to “give way”. In the present study, we derive the term from its use in locomotion, specifically referencing active contraction of leg extensors during weight acceptance [54]. More specifically, knee and ankle extensor contraction during flexion provides a buffer/cushion from impact forces and prevents destabilization [54]. Goslow and colleagues [55] definitively showed that *yield*-related muscle recruitment must be an *active* contractile element to be effective. Additionally, they demonstrated that, as the rate of locomotion increases, muscle recruitment during yield also increases in amplitude and duration.

During breathing, activity of diaphragm, parasternal, and external intercostal muscles steadily increases in amplitude to reach a maximum peak producing inspiratory airflow; this activity comprises the “I” phase (Fig 2) [56, 57]. The I phase is immediately followed by the beginning of the E phase (termed E1 or post-I in the literature [58, 59]), which starts with a decrementing diaphragm burst and is then followed by a period of diaphragm quiescence. Although the early phase of exhalation has traditionally been described as passive (i.e. no active abdominal contraction), there is indeed muscle activity from both diaphragm and expiratory laryngeal muscles. Rather than forcing air out of the lungs, these muscles activate to serve as a mechanical brake increasing laryngeal resistance and slowing exhalation (see review by Richter and Smith [60]). This regulation of expiratory airflow is also important in conditions where groups of various inspiratory, expiratory, pharyngeal, and laryngeal muscles must be precisely controlled. This tight regulation is evident during swallow, vocalization, and cough; in these situations, the lungs act as bellows to store air for the expulsion that is required for these motor behaviors. This phase has been called “E1”, “post-I”, “early-E”, and “E-dec”; here we are using the term “yield” to specifically refer to the active, cushioning properties (from remnant diaphragm, parasternal, and external intercostal activity) of this critical event.

It has recently been proposed that the post-inspiratory complex (PiCo) in the brainstem functions as a network oscillator to coordinate this phase of breathing with other central respiratory oscillators, and to produce state-dependent modulations as required for metabolic demands or precision motor acts [61, 62]. This is different and potentially adjunctive to laryngeal adduction, which has been classically used to define E1 [63–65]. Interestingly, in the current experimental preparation, the thyroarytenoid was active across the entire expiratory phase duration (Figs 1 and 2). This phenomenon of prolonged laryngeal adduction has also been demonstrated when breathing is at a mechanical disadvantage: infants, rodents (due to high lung compliance) [58], laparotomy [66], anesthetic states [67], and in cats anesthetized with chloralose-urethane [68] but not with sodium pentobarbital [68].

Our results also demonstrate that, during application of a mechanical challenge (chest compression), yield duration decreased in control conditions but increased following vagotomy, and there was no change in yield following pleural injection of lidocaine. We hypothesize that removal of all vagal feedback results in disinhibition, increasing yield duration. These results suggest that the yield phase is spinally mediated (possibly in part by intercostal proprioceptors rather than intrapleural sensors) and that the sum of vagal feedback tonically inhibits this component of breathing. Remmers [20] also concluded that supra-spinal inhibition and some spinal mechanism accounted for the response to chest compression.

This hypothesis of disinhibition with vagotomy has also been considered for respiratory control in general. Gautier [25] showed that removal of vagal feedback in one group of animals slowed RR due to prolonged I and E duration. Removal of spinal feedback via dorsal rhizotomy in another group of animals increased RR due to shortened I and E. When both vagal and spinal feedback were removed by bilateral vagotomy and dorsal rhizotomy, respectively, respiratory parameters did not significantly differ from those of intact animals [25], suggesting that breathing is properly maintained by mechanisms balancing vagal and spinal afferent feedback. This is consistent with experiments demonstrating that vagotomy and chest compression appear to have opposing effects on RR and inspiratory and expiratory durations [9–11, 18–21, 23, 25, 26].

### Laryngeal contribution to early-expiration (E1)

Interactions between the thyroarytenoid (laryngeal adductor) and respiratory pattern are modulated by vagally-mediated volume feedback onto adductor motoneurons [67]. Additionally, Bolser and Remmers [28] showed that stimulation of intercostal (thoracic) afferents depolarized expiratory vagal motoneurons, presumed to be laryngeal adductor motoneurons. Stimulation of intercostal mechanoreceptors also increased thyroarytenoid muscle activity [28]. When these receptors were stimulated during inspiration, thyroarytenoid motoneurons were activated; during expiration they were augmented [29]. In the current study, application of chest compression resulted in early excitation of laryngeal adduction during yield and altered muscle pattern activity throughout the expiratory phase (Fig 1). The activation of the thyroarytenoid muscles across the entire expiratory period limits further speculation about regulation of phase duration.

### Spinal afferent feedback

Lidocaine infused into the pleural space locally anesthetizes non-myelinated fibers of the peritoneum and the pleural space [69], as well as superficial mechano- and sensory receptors of the diaphragm, but has no effect on intercostal Golgi tendon organs and muscle spindles in the intercostal muscles (proprioceptors). Bolser et al. [30] showed that muscle/rib vibration inhibits inspiratory-related phrenic activity. This “inspiratory inhibitory reflex” is a result of activating intercostal Golgi tendon organs, rather than the muscle spindle endings that had been previously described [70]. Furthermore, thoracic dorsal rhizotomies (T1-T12) have effects on RR and lung volume that are believed to be caused by the loss of chest wall proprioception feedback onto medullary respiratory neurons [25, 71, 72]. During loading by tracheal occlusion, mechanoreceptors with afferent fibers in the cervical C3-C7 and thoracic T1-T9 regions are responsible for changes in medullary respiratory activity [73]. During chest compression, RR increases, and this response is eliminated by thoracic dorsal rhizotomy [74]. Collectively, this evidence indicates that characteristics of breathing can be strongly modulated by proprioceptive spinal afferent feedback.

Ramos [22], Campbell [12], Eccles, Sears, and Shealy [75] and their colleagues all suggested that thoracic cavity proprioceptive feedback significantly contributes to breathing patterns [12]. Three years later, Von Euler [76] proposed that intercostal muscle spindles act as a “follow-up length servo” by continuously adjusting muscle tension in response to volume demand. Using chest compression as a respiratory stimulant, the present study manipulated vagal and spinal feedback. Our results demonstrate that complete removal of vagal feedback in the presence of chest compression greatly influenced the yield subphase of expiration (Table 2A), and that removing pleura-related spinal feedback during chest compression influenced inspiration, expiration, and RR in females but not males (Table 2C). While pleural lidocaine administration produced some changes, at the low lung volumes produced by chest compression these changes may not be primary effectors of breathing pattern.

### Vagal feedback and sex as a biological variable

It has long been known that the vagus nerve contains PSR afferents [77–79], and that eliminating PSR feedback alters the breathing response to rapid lung inflation [25, 53, 80]. Studies by Cross et al. [50] in human and dog and by Fahim et al. [51] in cat demonstrated that aerosolized bupivacaine blocked the majority of PSRs, diminishing the Hering-Breuer inflation reflex. This, along with the lack of an inflation response in the present data, leads us to conclude that nebulization of 10% lidocaine reduced/eliminated PSR feedback. In the present experimental conditions, alteration of PSR-specific activity was not a significant contributor to the effects produced by chest wall constriction. However, vagotomy did produce a significant response, albeit sex-specific. The classic study by Gasser and Erlanger [81] suggested that non-specific local anesthetics first affect smaller and non-myelinated fibers. However, subsequent studies revealed that local anesthetics such as lidocaine have more nuanced effects and can produce a differential block of various sensory fiber types [82, 83]. Thus, it is possible that nebulized lidocaine also affected upper airway mucosal receptors other than PSRs. In one study, when lidocaine was injected into the pulmonary circulation, it inhibited airway mechanosensors [rapidly adapting and slowly adapting receptors (PSR)] but had an opposing stimulatory effect on airway chemoreceptors (C-fiber and high threshold Aδ-receptors) [84]. Inhaled lidocaine may selectively inhibit or stimulate particular airway vagal afferent types, while vagotomy would eliminate all vagal sensory information. We speculate that the differential effects of lidocaine on distinct vagal afferents may account for the differences in the results of nebulized lidocaine compared to those of bilateral vagotomy, and could also possibly contribute to the varying results in human inhaled lidocaine studies [33–35, 84].

In the present study, during control conditions, females had longer total respiratory cycle durations and reduced respiratory rates compared to males; due to an increase in late expiratory duration. This sex difference was enhanced with vagotomy, as late E duration was prolonged only in females, while both sexes had similar trends towards increases in I duration. There is limited information about sex differences in respiratory control, and many papers that present mixed animal groups do not specifically examine sex differences.

The sex difference we observed during chest compression may be due to thoracic geometry, chest wall compliance, or restriction band size relative to chest size. Alveoli of female rats are larger in quantity and smaller in size than those of males [85], resulting in larger alveolar surface area to body mass ratios in females compared to males [85, 86]. Male humans (on average) have a larger lung volume but a smaller volume to body mass ratio than females [86, 87]. Female humans have a smaller rib cage and a shorter diaphragm than males of the same height, and inspiratory intercostal muscles make a greater contribution to breathing in females compared to males [45]. Due to the inclination or angle of female ribs, the ribcage can accommodate greater volume expansion and increasing intercostal force compared to males [44, 45]. The result of this is that female breathing involves more thoracic contribution to movement, while male breathing involves more diaphragm contribution to movement [44, 45, 88]. We do not know if these anatomical differences are also present in the rat, but they do provide insight into possible causes for our observed sex differences during chest compression and afferent feedback manipulations.

### Limitations

Measures for female animals had a 243% increase in variability compared to males, as indicated by the standard deviation values throughout this study. This may be caused in part by the fact that the smallest and largest animals in this study were female (Fig 4), or by variations in estrus cycle, for which there were no controls. It may be important to compare effects across the estrus cycle or to use ovariohysterectomized animals in future work. Additionally, the number of female rats in our nebulized cohort was small due to animal death from cardio-respiratory failure. The nebulized lidocaine protocol was performed on 8 females, only 3 of which survived. The weight range of the animals that did not survive was 0.28kg to 0.43kg. The weight range of the animals that did survive was 0.34kg to 0.55kg. Based off their responsiveness to swallow stimulation and tail and toe pinch, male and female rats were at the same anesthetic state in this study, and they were given the same amount of anesthesia based on body weight. We speculate that the females were more sensitive to the nebulized lidocaine. The general anesthesia also has potential confounding effects. Known effects of sodium pentobarbital on gamma motoneurons could have reduced muscle spindle proprioceptive feedback in our animals. However, similar effects are reported in vagotomy studies using different anesthetics such as chloralose [53] and dial-urethane [89], and Adrian [53] concluded that anesthesia or decerebration had little impact on PSR activity.

## Conclusion

We propose that considering the E1 phase of breathing as a respiratory yield state could assist with interpreting differences in mechanistic descriptions of “post-I” activity versus “early-E” activity. The present data suggest that respiratory yield is strongly regulated by spinally-mediated proprioceptive (but not pleural) afferent feedback as a graded component of expiration. This has potential implications for individuals with spinal cord injuries involving thoracic levels, especially concerning their ability to produce robust responses to state-dependent respiratory challenges via local spinal circuits.

## Supporting information

S1 Data (XLSX)
